# Dissimilarities of neural representations of extinction trials are associated with extinction learning performance and renewal level

**DOI:** 10.3389/fnbeh.2024.1307825

**Published:** 2024-02-26

**Authors:** Silke Lissek, Martin Tegenthoff

**Affiliations:** Department of Neurology, BG University Hospital Bergmannsheil, Ruhr-University Bochum, Bochum, Germany

**Keywords:** extinction, renewal, representational similarity analysis, hippocampus, vmPFC, IFG

## Abstract

**Introduction:**

Renewal of extinguished responses is associated with higher activity in specific extinction-relevant brain regions, i.e., hippocampus (HC), inferior frontal gyrus (IFG), and ventromedial PFC (vmPFC). HC is involved in processing of context information, while IFG and vmPFC use such context information for selecting and deciding among competing response options. However, it is as yet unknown to what extent trials with changed versus unchanged outcome, or extinction trials that evoke renewal (i.e., extinction context differs from acquisition and test context: ABA trials) and trials that do not (i.e., same context in all phases: AAA trials) are represented differentially in extinction-relevant brain regions.

**Methods:**

In this study, we applied representational similarity analysis (RSA) to determine differences in neural representations of these trial types and their relationship to extinction error rates and renewal level.

**Results:**

Overall, individuals with renewal (REN) and those without (NoREN) did not differ significantly in their discrimination levels between ABA and AAA extinction trials, with the exception of right posterior HC, where REN exhibited more pronounced context-related discrimination. In addition, higher dissimilarity of representations in bilateral posterior HC, as well as in several IFG regions, during extinction learning was linked to lower ABA renewal rates. Both REN and NoREN benefitted from prediction error feedback from ABA extinction errors for context- and outcome-related discrimination of trials in IFG, vmPFC, and HC, but only the NoREN group also benefitted from error feedback from AAA extinction errors.

**Discussion:**

Thus, while in both groups the presence of a novel context supported formation of distinct representations, only in NoREN the expectancy violation of the surprising change of outcome alone had a similar effect. In addition, only in NoREN context-related discrimination was linked to error feedback in vmPFC. In summary, the findings show that context- and outcome-related discrimination of trials in HC, vmPFC, and IFG is linked to extinction learning errors, regardless of renewal propensity, and at the same time point towards differential context processing strategies in REN and NoREN. Moreover, better discrimination of context-related trials during extinction learning promotes less renewal during extinction recall, suggesting that renewal may be related to suboptimal context-related trial discrimination.

## 1 Introduction

Extinction learning and renewal recruit a network of brain regions comprising, among others, ventromedial prefrontal cortex (vmPFC), inferior frontal gyrus (IFG), and hippocampus (HC) (Kalisch et al., 2006; Milad et al., 2007; Lissek et al., 2013, 2020). The term renewal describes the recovery of extinguished responses when recall is performed in a context that differs from the context present during extinction learning (Bouton and Bolles, 1979). Therefore, renewal illustrates the context-dependency of extinction learning. Renewal has predominantly been researched for fear extinction, but the effect is also regularly observed during non-fear related extinction learning, such as appetitive extinction learning or causal learning (e.g., Bouton and Peck, 1989; Vila and Rosas, 2001; Üngör and Lachnit, 2006; Üngör and Lachnit, 2008). Studies of non-fear related extinction learning show that individuals differ in their propensity for renewal, and that their behavior is consistent over repeated sessions of the task. Thus, showing or not showing renewal appears to constitute a stable processing strategy (Uengoer et al., 2020).

Individuals with (REN) and without a propensity for renewal (NoREN) differ regarding their level of activation in several regions of the extinction-related network. REN individuals exhibit stronger blood-oxygen-level-dependent (BOLD) activation in HC, vmPFC, and IFG during extinction learning proper and recall of extinction memory (e.g., Lissek et al., 2013, 2016, 2020). It is assumed that HC contributes to processing of context during extinction learning and delivers context information during retrieval of extinction memory. HC activation during extinction learning and recall/renewal is observed in anterior as well as posterior regions, with inconclusive evidence regarding potentially specific contributions of these subregions (e.g., Kalisch et al., 2006; Milad et al., 2007; Garfinkel et al., 2014; Hermann et al., 2016). It has been proposed that in general, anterior HC represents rather global, generalized information, and posterior HC rather fine-grained and detailed information (Poppenk et al., 2013; Schlichting et al., 2015). A recent study found stronger connectivity of anterior HC with vmPFC and of posterior HC with IFG (Frank et al., 2019).

Ventromedial PFC is recruited during extinction learning and recall in REN individuals (Lissek et al., 2013, 2016, 2020), thus is presumably active in selecting the proper response considering context information. Also, vmPFC has been shown to be involved in reward and value-based decision making (Rolls, 2022) in various tasks. Activity in vmPFC during extinction learning in a novel context was found positively correlated with ABA renewal levels, suggesting that higher BOLD activation during encoding of new assocations resulted in a better assignment of rewards to their respective learning phase contexts (Lissek et al., 2015a,b).

Regions in IFG also show stronger BOLD activation during extinction learning and recall in REN individuals (Lissek et al., 2020). It is assumed that IFG activation reflects response inhibition (Konishi et al., 1999; Swick et al., 2008; Hampshire et al., 2010) and/or response selection from competing response options under conditions of ambiguity (Budhani et al., 2007; Mitchell et al., 2009). Thus, IFG-mediated response selection in contextual extinction tasks is presumably necessary when the contexts are considered in which the respective stimulus-outcome association was formed.

Therefore, IFG and vmPFC presumably cooperate in deciding upon the proper response, with IFG providing information about competing response options and vmPFC providing reward-related information.

While the brain regions which show renewal-related activation differences are largely established, it is as yet unclear how these regions process the neural representations of extinction learning trials. In a typical extinction task designed to evoke renewal, two basic types of trials are compared: trials with extinction learning in a novel context (ABA condition) and trials with extinction learning in the context of acquisition and test (AAA condition). It is assumed that renewal occurs when the surprising change of outcome during extinction trials provokes increased attention to the context (Darby and Pearce, 1995), so the novel context is linked to the changed stimulus-outcome association. Therefore, trials with and without a novel context in the extinction phase will probably be represented differentially in extinction-related brain regions.

Moreover, presumably there will be a relationship between behavioral measures of extinction and the distinctness of representations. A higher rate of extinction learning errors may support the differentiation between trial types with and without a novel context, since more errors provide more instances of increased attention to relevant characteristics of the task environment, such as the context. Also, more distinct representations of trial types may influence renewal, since a better discrimination between trials with and without a novel context may reduce the ambiguity of competing response options and thus support choosing the option considered as correct.

Since basically, extinction learning does not erase initial learning, but involves the generation of a new inhibitory association (Bouton, 2004) that competes with the existing association, neural representations of trials whose outcome has changed versus whose outcome remains unchanged will also differ. During extinction learning, wrong responses will result in a prediction error signal (Rescorla and Wagner, 1972) regarding the outcome. Therefore, possibly also the differentiation between trial types with and without a changed outcome, containing extinguished and unextinguished stimuli, respectively, will be related to errors made during extinction.

A method for investigation of differences in brain representations is the representational similarity analysis (RSA) (Kriegeskorte et al., 2008). By means of RSA, the brain activity patterns (“representations”) for pairs of experimental conditions are compared by spatial correlation, yielding dissimilarity values (DVs) for each pair in terms of correlation distance. By computing representational dissimilarity matrices (RDMs), the dissimilarity between the activity patterns associated with the two conditions can be visualized. The higher the DV, the less similar are the representations of the two conditions. RSA has been used in a number of studies to investigate, among other topics, concept processing (Carota et al., 2021), responses to CS+ in fear extinction learning (Graner et al., 2020), or processing of different contextual features in the developing HC (Kazemi et al., 2022).

In our study, we used RSA to determine to what extent REN and NoREN participants differ with regard to the dissimilarity of their representations of trials which display either the same or a novel context (during the extinction phase), as well as trials in which the outcome changes or does not change, and how such dissimilarities are related to their learning and renewal performance. For the analysis, we used datasets from a non-fear related extinction task, a so-called predictive learning task. In this task, typically only a certain percentage (45–65%) of participants exhibit a renewal effect (e.g., Lissek et al., 2013, 2016), which makes the task particularly suited for the investigation of differences in processing between REN and NoREN individuals, since opposing responses to an identical input can be analyzed.

We assumed that differences in context-related neural representations between the groups would appear in particular in HC, iFG, and vmPFC, since these regions were previously shown involved in processing and evaluation of context information in extinction learning, with higher activation in REN individuals. In particular, we expected more dissimilar representations of trials with and without a novel context in REN, compared to NoREN, in the ABA recall phase, in which REN shows renewal. Moreover, we expected a positive relationship between dissimilarity of these representations and ABA extinction errors, respectively ABA renewal.

Furthermore, we analyzed potential differences regarding dissimilarity of representations of trials that changed or did not change their outcome during the extinction phase, i.e., contained extinguished or unextinguished stimuli. Here, we investigated to what extent the selected brain regions also process the altered consequence in extinction proper. We assumed that the analyzed regions involved in extinction learning would display differences in outcome-related neural representations in both groups alike, and that these differences would be related to extinction errors.

## 2 Materials and methods

### 2.1 Participants

109 fMRI datasets were included in the RSA analysis. The datasets derive from previous fMRI studies, that investigated context-related extinction learning and renewal and used the same version of a predictive learning task (mainly from Lissek et al., 2019, 2020, 2022). Prerequisites for datasets selected to be included in the analysis were: high BOLD imaging data quality (i.e., discernible activation) for the extinction and recall phase of the task in the extinction-relevant brain regions considered in the RSA analysis, placebo treatment or no treatment. Inclusion criteria for participation in the original study were: 18–40 years of age, no current medical or neurological condition, right-handedness, and normal vision. Based on the ABA renewal level identified in the respective studies, each dataset was assigned to one of these two groups: REN (renewal *n* = 49) and NoREN (no renewal *n* = 60). Criteria for assignment to the REN group were ≥ 20% ABA renewal responses during the recall phase. Datasets with <20% ABA renewal responses were assigned to the NoREN group.

The datasets come from placebo control subjects to pharmacological treatments (*n* = 65), of which two studies have been published (Lissek et al., 2019, 2022), and from the sample of a study using context modulation (*n* = 44) (Lissek et al., 2020). The total sample consisted of 53 males and 56 females (control subjects 31 males and 34 females, context subjects 22 males and 22 females). In the controls group, 28 subjects showed renewal while 37 did not, in the context group 21 subjects showed renewal while 23 did not.

### 2.2 Ethics statement

The studies underlying this analysis conformed to the Ethics of the Word Medical Association (Declaration of Helsinki) and were approved by the local ethics board of the Ruhr University Bochum (Reg.No. 3022–10 dated 11.02.2016). All subjects participated after giving written informed consent.

### 2.3 Predictive learning task

The present study used the same or similar methods as in a number of our prior publications (among others; Lissek et al., 2013, 2015a,b, 2016). Therefore, we are using similar text for the task descriptions.

The studies from which the fMRI datasets were derived applied a predictive learning task originally designed by Üngör and Lachnit (2006), to investigate associative extinction learning and the context-related renewal effect without a fear component. In the predictive learning task, participants learn to associate several stimuli/cues (food items) with particular consequences (occurrence or non-occurrence of a stomach ache) in different contexts (restaurants). They do so by taking the role of a physician who has to predict whether their patient will develop a stomach ache after eating certain foods. The learning process consists of the three successive phases of (a) acquisition of associations, (b) extinction phase, and (c) test/recall phase (see Table 1). In the extinction phase, previously acquired associations between an item of food and stomach ache are extinguished.

**Table 1:** Task design of the predictive learning task (note that the classification of stimuli into extinction, retrieval, and new learning stimuli only applies from the extinction phase on).

Condition		Acquisition		Extinction		Test	
		Context 1	Context 2	Context 1	Context 2	Context 1	Context 2
AAA	Extinction	A+	B+	A−	B−	A?	B?
	Retrieval	C+	D−	C+	D−	C?	D?
	New learning	I−	J−	K+	L+		
		Q−	R+				
ABA	Extinction	E+	F+	F−	E−	E?	F?
	Retrieval	G+	H−	H−	G+	G?	H?
	New learning	M−	N−	P+	O+		
		S−	T+				

Extinction stimuli and retrieval stimuli appeared in all learning phases. New learning stimuli appeared only in the respective learning phase to balance the design. Overall, + and – signs after the stimuli letters indicate whether the stimulus signalled stomach ache (= CS+) or no stomach ache (=CS−).

Learning takes place in two conditions: (a) extinction learning occurs in a context different from that present during acquisition and recall (ABA) and (b) all learning phases occur in the same context (AAA). The task contains *extinction trials*, in which the consequence of stomach ache changes in the extinction phase (ABA CC/AAA CC trials). The task also contains *retrieval trials*, in which the consequence of stomach ache does not change during the extinction phase (ABA nonCC/AAA nonCC)—providing a control of acquisition learning success and the option to compare changed/unchanged consequence trials during extinction, either with a novel or identical context (see Table 1).

**Figure 1:**
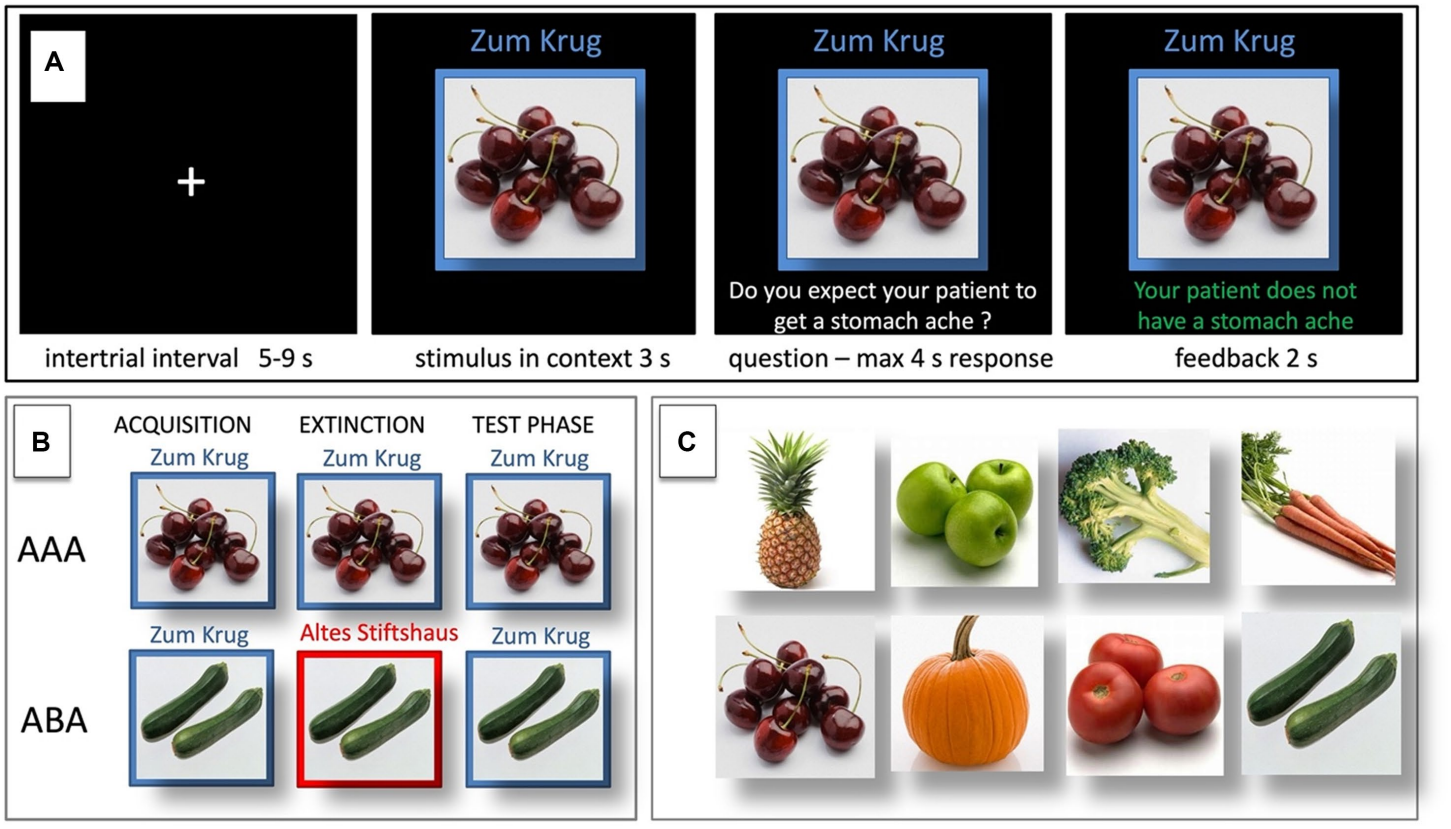
Predictive learning task. **(A)** Example of a single trial. **(B)** ABA and AAA conditions, showing the context change in ABA. **(C)** Examples of food stimuli.

During the acquisition phase, participants learn to associate a presented food item with a consequence. In each trial, a stimulus (photo of a vegetable or a fruit) is presented to the participant in one of two available contexts. The contexts consist of the restaurant names “Zum Krug” (The Mug, 1) and “Altes Stiftshaus” (The Dome, 2) and a frame in either red or blue color (see Figure 1).

First, the stimulus in its context is presented for 3 s, then a question asking whether the patient will develop a stomach ache is superimposed on the frame, together with the response options “Yes” or “No.” Participants respond by pressing the respective button on an fMRI-ready keyboard (Lumitouch, Photon Control Inc., Richmond, BC, Canada) within a time window of 4 s. After the response, else after expiration of the response time, feedback with the correct answer is displayed for 2 s, i.e., “The patient has a stomach ache” or “The patient does not have a stomach ache.” The actual response of the participant is not commented upon.

The food stimuli are presented in randomized order. The acquisition phase contains 16 different stimuli, eight stimuli per context. Each stimulus is presented eight times, amounting to a total of 128 trials. Half of the stimuli predict stomach ache, the others predict no stomach ache. The consequence of stomach ache is counterbalanced to appear equally often in both contexts.

During the extinction phase, half of the stimuli from the acquisition phase (8) are presented again. Of these, one half (4) is presented in the same context as during acquisition (condition AAA—no context change) and the other half (4) in a different context (condition ABA—context change) in randomized order. Within these groups of stimuli, a further distinction is being made between actual extinction stimuli (i.e., stimuli for which the consequence of stomach ache changes to no stomach ache during extinction) and retrieval stimuli (for which the consequence of stomach ache does not change), resulting in two extinction stimuli and two retrieval stimuli per context. Also, four new stimuli are introduced during the extinction phase, to balance the design so that it contains equal numbers of stimuli predicting stomach ache in both contexts. Overall, thus, the extinction phase contains a total of 12 different stimuli, six per context, i.e., two extinction stimuli, two retrieval stimuli, and two new stimuli per context. Each stimulus is being presented eight times, amounting to a total of 96 trials. Again, half of the stimuli predict stomach ache, the others predict no stomach ache, and the consequence of stomach ache is counterbalanced to appear equally often in both contexts. In all other respects, trial design is identical to acquisition. Also, during all trial types in the extinction phase, participants receive feedback on the correctness of their response.

During the recall phase, extinction and retrieval stimuli are presented once again in the context of acquisition (five presentations per stimulus), resulting in a total of 40 trials. With the exception that during the recall phase participants receive no feedback with the correct response, trials are identical to those during acquisition.

For a detailed overview of the stimulus types, task phases, and context conditions, please refer to Table 1 and Figure 1.

### 2.4 Imaging data acquisition

Functional and structural brain scans were acquired using a whole-body 3 T scanner (Philips Achieva 3.0 T X-Series, Philips, The Netherlands) with a 32-channel SENSE head coil. Blood-oxygen level dependent (BOLD) contrast images were obtained with a dynamic T2* weighted gradient echo echoplanar imaging (EPI) sequence using SENSE (TR 3,200 ms, TE 35 ms, flip angle 90°, field of view 224 mm, slice thickness 3.0 mm, and voxel size 2.0 mm × 2.0 mm × 3.0 mm). We acquired 45 transaxial slices parallel to the anterior commissure—posterior commissure (AC-PC) line, which covered the whole brain. High resolution structural brain scans of each participant were acquired using an isotropic T1 turbo-field echo (TFE) sequence (field of view 240 mm, slice thickness 1.0 mm, voxel size 1 mm × 1 mm × 1 mm) with 220 transversally oriented slices covering the whole brain. The task was presented to the participants via fMRI-ready liquid-crystal display (LCD) goggles (Visuastim Digital, Resonance Technology Inc., Northridge, CA, United States) connected to a laptop which ran specific software programmed in Matlab. Responses were given by means of an fMRI-ready keyboard (Lumitouch response pad, Photon Control Inc., Canada).

### 2.5 Imaging data analysis

For preprocessing and statistical analysis of fMRI data, we used the software Statistical Parametric Mapping (SPM), Version 12 (Wellcome Department of Cognitive Neurology, London, United Kingdom), implemented in Matlab R2017b (Mathworks, Natick, MA, United States). Three dummy scans, during which the BOLD signal reached steady state, preceded the actual data acquisition of each session. Thus preprocessing started with the first acquired volume. Preprocessing on single subject level consisted of the following steps: slice timing correction to account for time differences due to multislice image acquisition; realignment of all volumes to the first volume for motion correction; spatial normalization into standard stereotactic coordinates with 2 × 2 × 2 mm^3^ using an EPI template of the Montreal Neurological Institute (MNI) provided by SPM, smoothing with a 6 mm full-width half-maximum (FWHM) kernel, in accordance with the standard SPM procedure. The acceptable limit for head motion was 2 mm for translational movements and 0.5° for rotational movements.

In a first level single subject analysis we calculated activation during acquisition, extinction and test (recall) phases for ABA and AAA conditions, as well as conditions with and without change of outcome, contrasted against baseline. The respective beta maps from these single subjects analyses were entered into the RSA analysis.

### 2.6 Representational similarity analysis

The Representational Similarity Analysis (RSA) was performed using the rsatoolbox (https://github.com/rsagroup/rsatoolbox; Nili et al., 2014) written for Matlab (The Mathworks, Inc., Natick, MA, United States). RSA uses the beta values from the first-level analysis of the single subjects for the relevant contrasts.

By means of RSA, we estimated the level of dissimilarity between the neural patterns evoked by trials that use a novel context during extinction (ABA), and those that use the same context during extinction (AAA) as was present during acquisition and recall. Furthermore, we estimated the level of dissimilarity between extinction and retrieval trials performed in the same condition (i.e., in either ABA or AAA).

**Figure 2:**
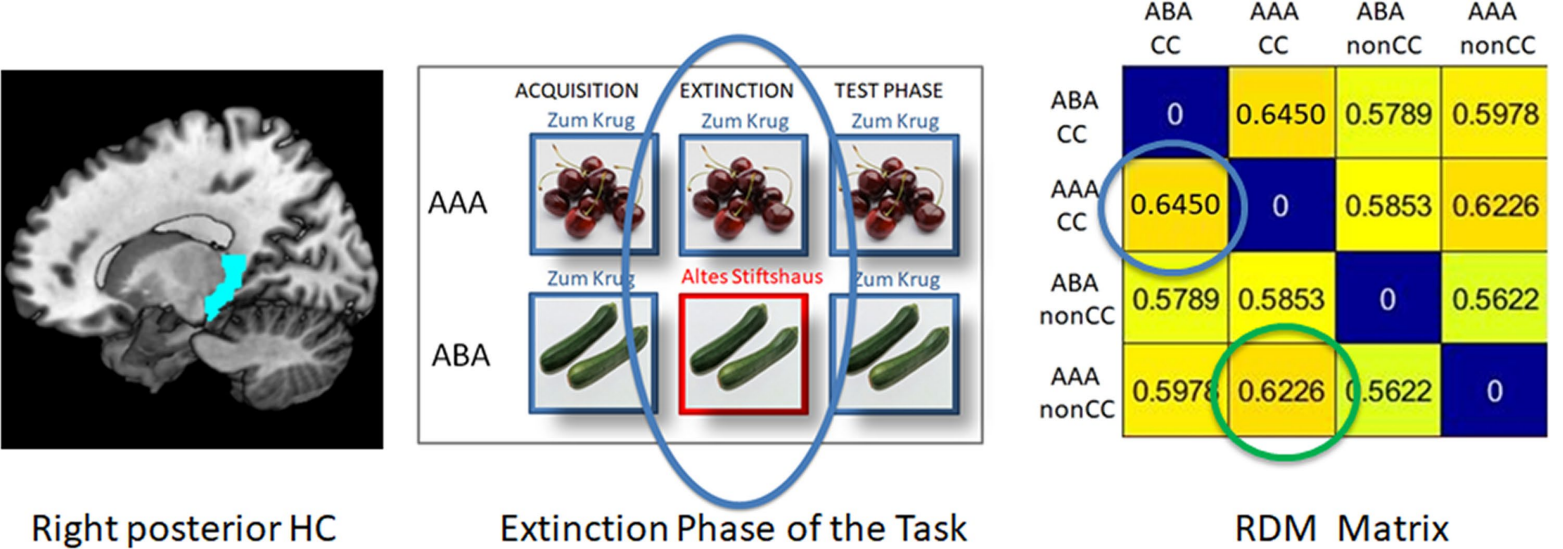
Example of an RDM matrix of the extinction learning phase (here: right posterior HC, REN group). The blue circle in the RDM matrix contains the context-related dissimilarity value between ABA CC and AAA CC, i.e., extinction trials with a novel vs. the identical context. The green circle shows the outcome-related dissimilarity value between AAA CC and AAA nonCC, i.e., the comparison between an extinction trial and a retrieval trial in which the outcome does not change.

In each single subject dataset, a Representational Dissimilarity Matrix (RDM) was calculated for the extinction and retrieval trials in the ABA and AAA conditions during the extinction and recall phases for the pre-selected regions of interest (ROIs). This RDM yields a dissimilarity value (DV) for each combination of these conditions (see example in Figure 2).

By means of RSA, to produce the RDM, the correlation between the values of the conditions is calculated. The resulting DV contained in the RDM is 1 minus the calculated correlation value (Pearson’s correlation coefficient). If the neural patterns are similar for the compared conditions, their correlation will be high and thus the DV will be low. In contrast, if the neural patterns are rather different, a low correlation will result, which again produces a high DV.

In case of the predictive learning task, DVs can be calculated to investigate context-related differences between trial types, by comparing trials containing a novel context (ABA) or the same context (AAA) during the extinction phase. Also, DVs can be calculated to investigate outcome-related differences between trial types, by comparing trials that contain an unchanged outcome (nonCC) or a changed outcome (CC) during the extinction phase (i.e., retrieval and extinction trials). In both cases, the DV between the trial types presumably reflects the specific difference that exists between the trials, i.e., either a change/no change of context and/or of outcome. We restricted our further analysis to comparisons of trials in which only one of these factors differed, either the context or the outcome (see Figure 1).

The group means of relevant DVs were compared between REN and NoREN groups to determine extinction-related brain regions in which the DVs differed between participants with and without a propensity for renewal, and which may thus be involved in processing of context-related information. Moreover, we calculated correlations between context-related DVs and performance results (i.e., ABA extinction errors, ABA renewal) in order to determine whether the ability to discriminate between the respective trial types in terms of the context was associated with the error rate in ABA trials during extinction learning and with the resulting ABA renewal level during recall. In addition, correlations between outcome-related DVs, that address the differentation of extinguished versus unextinguished stimuli, and performance results were calculated.

Representational similarity analysis was performed for the a priori ROIs: HC (anterior/posterior), IFG (triangular BA 45, opercular BA 44, and orbital BA 47), and vmPFC (BA 10/11). The anatomical ROIs were defined selecting the respective regions from the WFU pickAtlas Toolbox implemented in SPM12 (Tzourio-Mazoyer et al., 2002). The anterior and posterior HC ROIs were defined by separating the HC mask at the level of *Y* = −21 mm, using the uncal apex as a landmark for HC segmentation (see Poppenk et al., 2013).

### 2.7 Behavioral and RSA data analysis

We calculated the number of extinction errors in ABA and AAA trials in the extinction learning phase, as well as the level of ABA renewal during the recall phase, to be used in the RSA analysis reported here.

The data were derived from log files written for all three learning phases, which contained information on response latency, response type, and correctness of response, from which we calculated error rates during extinction learning. For calculation of the renewal level, during the recall phase only responses to stimuli with consequence change (extinction stimuli) were analyzed. The behavioral renewal effect in the predictive learning task is supposed to occur only in the condition ABA, due to the context change introduced during extinction learning. In case of renewal, associations learned during acquisition in context A will reflect in responses during the test phase, which is again performed in context A, while extinction was performed in context B. In contrast, the AAA condition constitutes a control condition for extinction learning, since here all learning phases are performed in an identical context. If extinction learning is successful, responses during the test phase will reflect the associations learned during the extinction phase. However, if extinction learning is impaired, responses in the AAA test phase may reflect associations learned during acquisition.

Errors in acquisition and extinction learning were defined as responses stating the incorrect association between the context-cue-compound and the consequence.

During the recall phase, a response that referred to the association which was correct during acquisition constituted an error in the AAA condition, and a renewal response in the ABA condition.

Statistical analyses (*t*-tests, correlations) were performed using the software package IBM SPSS Statistics for Windows, version 27.0 (IBM Corp, Armonk, NY, United States). Correction for multiple comparisons at a threshold of *p* < 0.05 was performed, where applicable, using the method stipulated by Benjamini and Hochberg (1995). Correlations (Pearson’s correlation coefficient) are reported corrected for multiple comparisons as stated above; one-tailed for directional hypotheses such as concerning the relationship between DVs and learning performance, and two-tailed for non-directional hypotheses, as in the analyses of individuals with higher and lower renewal levels.

## 3 Results

### 3.1 Comparisons of context-related and outcome-related DVs between REN and NoREN groups

#### 3.1.1 Comparison of REN and NoREN groups for context-related DVs (extinction trials performed in an identical or novel context—ABA CC vs. AAA CC)

**Figure 3:**
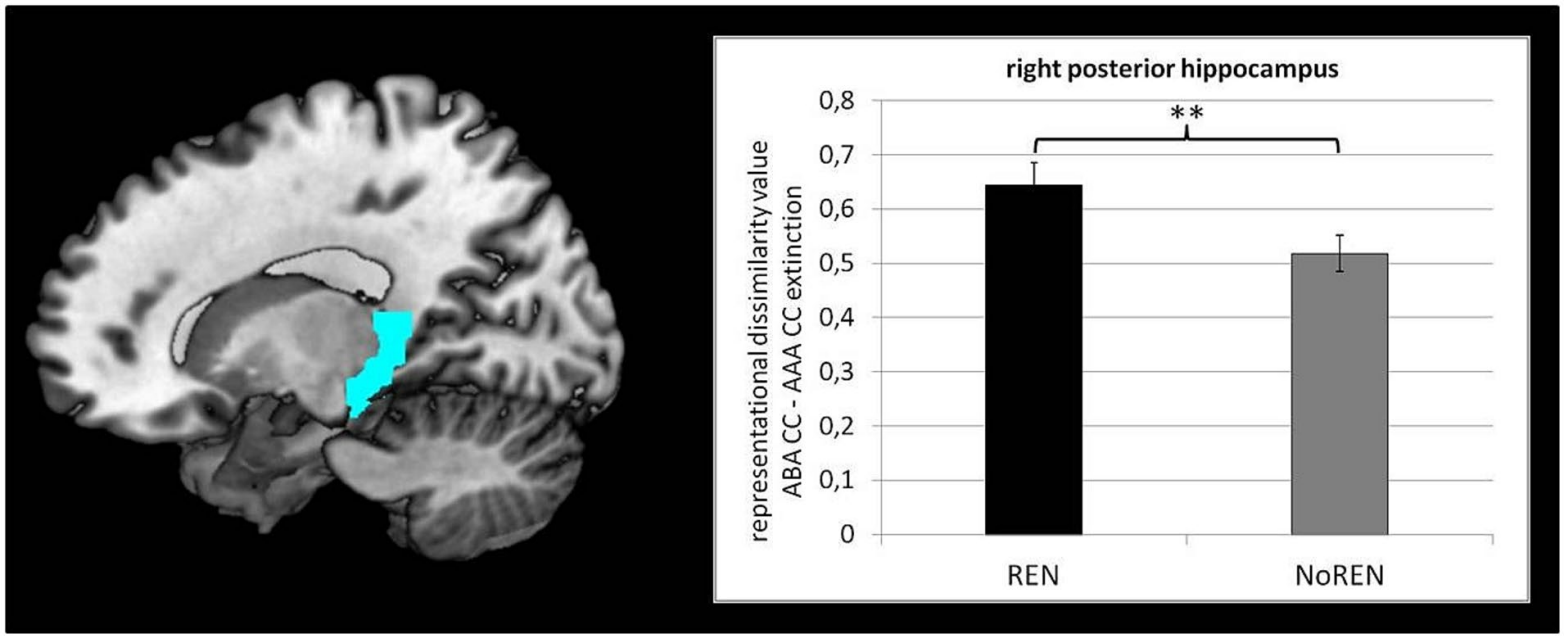
Significant differences between REN and NoREN in context-related DVs of ABA CC–AAA CC extinction trials in right posterior HC [*t*(107) = 2.415, *p* = 0.008; one-tailed].

To test our hypothesis of larger context-related DVs in the REN group, we compared the DVs of ABA CC—AAA CC extinction trials of the REN and NoREN groups in anterior/posterior HC, orbital, opercular, and triangular iFG, as well as vmPFC, respectively, by means of two-sample tests. Significant differences were found in right posterior HC only [*t*(107) = 2.415 *p* = 0.008 one-tailed] [mean DVs REN 0.6450 (± 0.041 s.e.m.)], NoREN 0.5181 (± 0.034 s.e.m.) (see Figure 3).

In contrast, the DVs of ABAnonCC—AAA nonCC retrieval trials during the extinction phase did not differ significantly between the groups in HC.

Furthermore, in other regions (IFG and vmPFC), there were no significant differences in DVs between the groups.

#### 3.1.2 Comparison of REN and NoREN groups for outcome-related DVs (trials with changed and unchanged outcomes—CC vs. nonCC)

In addition, using two-sample tests, we compared DVs of trials with a changed or unchanged outcome (CC and nonCC), in the two conditions of either identical or novel extinction context (ABA or AAA) during extinction and recall. A non-significant difference (two-tailed test after correction for multiple comparisons) was found in right BA 45 for AAA CC–AAA nonCC recall trials—here the DVs of the NoREN group were higher than in the REN group [*t*(107) = 2.173, *p* = 0.032]. Also, none of the other regions showed differences between the groups.

### 3.2 Correlations

#### 3.2.1 Correlations of ABA extinction errors with context-related DVs for ABA CC–AAA CC in extinction and recall phases

All significant correlations between ABA extinction errors and context-related DVs were positive, indicating that in both groups a high error level supported better discrimination of trials with or without a novel context.

Both REN and NoREN groups exhibited positive correlations of ABA extinction errors with discrimination values for trials in a novel context compared to the identical context (DVs for ABA CC–AAA CC) during recall in bilateral BA 47 (orbital IFG).

In addition, in the REN group only, positive correlations with ABA extinction errors were found for DVs in bilateral BA 45 (triangular IFG) and right BA 44 (opercular IFG) during recall, and for left posterior HC during extinction learning proper. In NoREN only, significant positive correlations of DVs for ABA CC–AAA CC in left posterior HC as well as in bilateral vmPFC were found during the recall phase.

Taken together, thus, compared to NoREN, the REN group shows additional IFG regions in which ABA extinction errors appear to drive the discrimination performance between ABA and AAA trials: namely bilateral triangular IFG and right opercular IFG. In bilateral orbital IFG, in contrast, ABA extinction errors influence the discrimination level in both groups, regardless of their renewal propensity. In contrast, in NoREN, bilateral vmPFC figures prominently in the discrimination between ABA and AAA trials.

**Figure 4:**
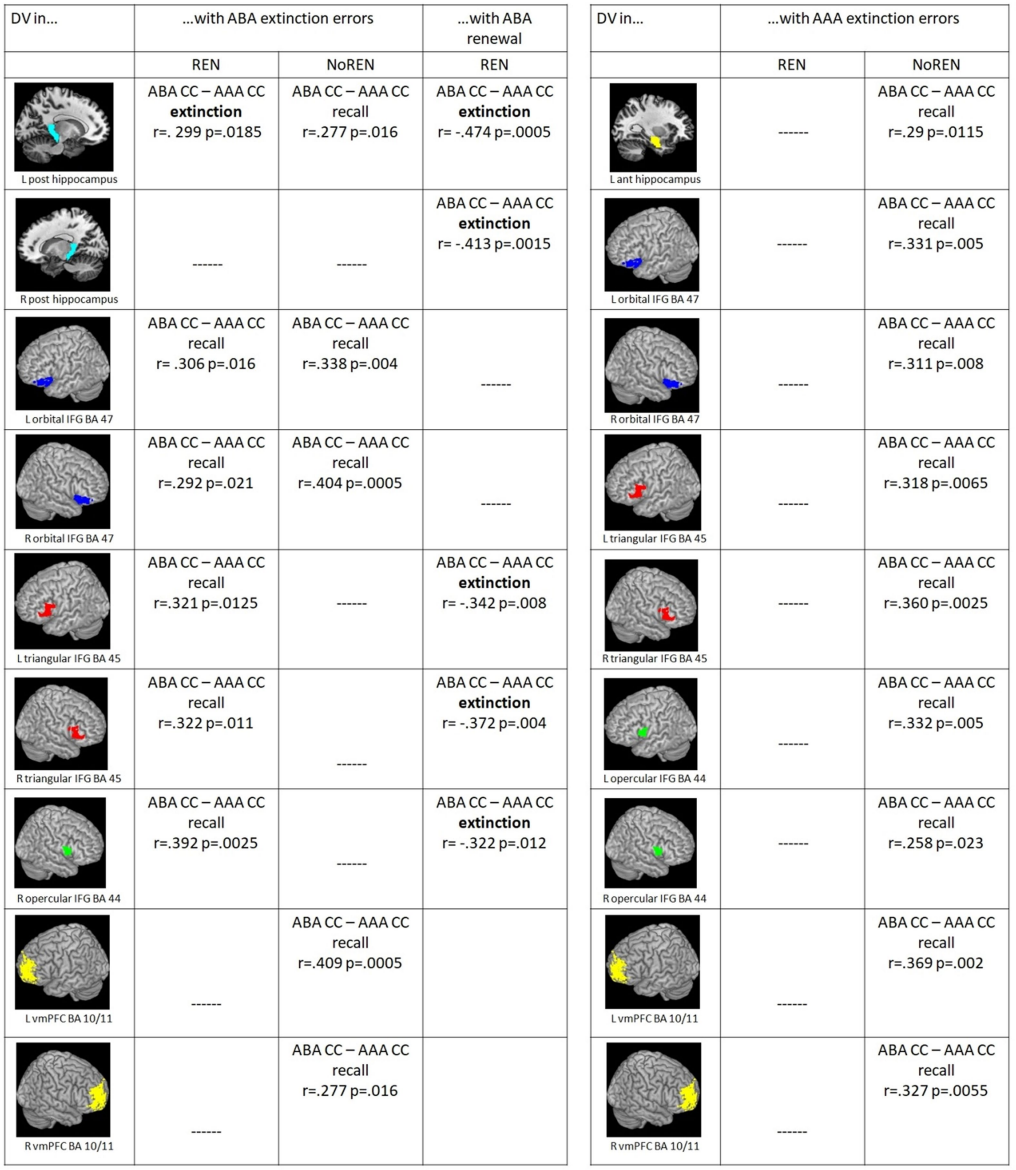
**(Left)** Significant correlations between ABA extinction errors and context-related DVs of ABA CC–AAA CC extinction trials/recall trials in the REN and NoREN groups, as well as significant correlations between ABA renewal level and context-related DVs of ABA CC–AAA CC extinction trials in the REN group (Pearson’s correlation coefficient, one-tailed). **(Right)** Significant correlations between AAA extinction errors and context-related DVs of ABA CC–AAA CC recall trials in the REN and NoREN groups (Pearson’s correlation coefficient, one-tailed).

Furthermore we found a dissociation of context-related (ABA CC–AAA CC) DVs in left posterior HC pertaining to the learning phase in which significant correlations with ABA extinction errors were observed: ABA extinction errors support better discrimination between trial types in left posterior HC already during extinction learning proper in REN, while in NoREN this extinction-error driven better discrimination occurred only in recall (see Figure 4).

#### 3.2.2 Correlations of AAA extinction errors with context-related DVs for ABA CC–AAA CC in recall

In a further analysis, we calculated correlations of AAA extinction errors with context-related discrimination values for trials in a novel context compared to the identical context (ABA CC–AAA CC). Here, the REN group showed no significant correlations at all, indicating that AAA extinction errors did not support their context-related discrimination performance. In contrast, in the NoREN group, significant positive correlations were observed for all three bilateral IFG regions as well as for bilateral vmPFC, and for left anterior HC. The data suggest a different processing focus of the NoREN group, in which expectancy violations brought about by AAA extinction errors (i.e., errors in extinction trials without a novel context) contribute pronouncedly to context-related discrimination, in contrast to REN, in which they play no role at all for this discrimination (see Figure 4).

#### 3.2.3 Correlations of the ABA renewal level with context-related DVs for ABA CC–AAA CC during the extinction phase

For ABA renewal, correlations with discrimination values were calculated only for the REN group, since the NoREN group exhibited, by definition, no ABA renewal. In pronounced contrast to ABA extinction errors, all correlations of ABA renewal with discrimination values for trials in a novel context compared to the identical context (ABA CC–AAA CC) in HC and IFG were negative, indicating that a better discrimination between ABA and AAA trials during the extinction phase was associated with a lower level of ABA renewal during the subsequent recall phase. This result was found for bilateral posterior HC, bilateral triangular IFG and right opercular IFG (see Figure 4).

Thus, in the REN group, discrimination values in the same IFG regions correlated (a) positively during recall with the preceding ABA extinction errors, (b) negatively during extinction learning with the subsequent ABA renewal level, indicating that the higher ABA extinction error level increased differentiation ability in recall, while lower discrimination ability during extinction was linked to more renewal in recall.

To check whether these negative correlations occurred predominantly in individuals with higher or lower ABA renewal levels, we subdivided the REN group into two subgroups: (a) a lower ABA renewal level (20–60%, *n* = 25 RENlow mean: 45.6% ± 2.39) and (b) a higher ABA renewal level (70–100%, *n* = 24 RENhigh, mean: 91.25% ± 2.28). Separately for these two groups, we calculated again those correlations that were significant in the complete REN group. Since we had no directional hypotheses here, test results are reported two-tailed. Importantly, for right posterior HC, right opercular IFG and bilateral triangular IFG, significant negative correlations were observed only for the RENhigh group. Only in left posterior HC, the negative correlations were significant for both subgroups (see Table 2).

**Table 2:** Correlations of ABA renewal with DVs from ABA CC to AAA CC extinction in REN participants with high (70–100%) and low (20–60%) renewal levels (Pearson’s correlation coefficient, two-tailed).

	RENhigh	RENlow
Left posterior HC	*R* = −0.492, *p* = 0.015	*R* = −0.438, *p* = 0.028
Right posterior HC	*R* = −0.580, p = 0.003	*R* = −0.381, *p* = 0.060
Right opercular IFG BA 44	*R* = −0.598, *p* = 0.002	*R* = −0.384, *p* = 0.058
Left triangular IFG BA 45	*R* = −0.742, *p* = 0.000	*R* = −0.307, *p* = 0.136
Right triangular IFG BA 45	*R* = −0.436, *p* = 0.033	*R* = −0.229, *p* = 0.272

To illustrate the difference between the groups, here also the non-significant correlations are listed.

The result shows that the negative correlations of ABA renewal levels with context-related DVs depend largely on the behavior patterns of participants with a higher level of renewal.

#### 3.2.4 Correlations of the ABA renewal level with context-related DVs for ABA CC–AAA CC in the recall phase

In addition, negative correlations between ABA renewal and context-related DVs of ABA and AAA trials *during the recall phase* were observed in different regions, however, they did not survive the threshold for multiple comparisons (*p* < 0.05). In right anterior HC (*r* = −0.250, *p* = 0.085 two-tailed), left BA 47 (*r* = −0.249, *p* = 0.085 two-tailed), and right BA 47 (*r* = −0.241, *p* = 0.095 two-tailed), context-related DVs correlated negatively with ABA renewal level.

#### 3.2.5 Correlations of outcome-related DVs (ABA CC–ABA nonCC, AAA CC–AAA nonCC) with ABA or AAA extinction errors, respectively

**Figure 5:**
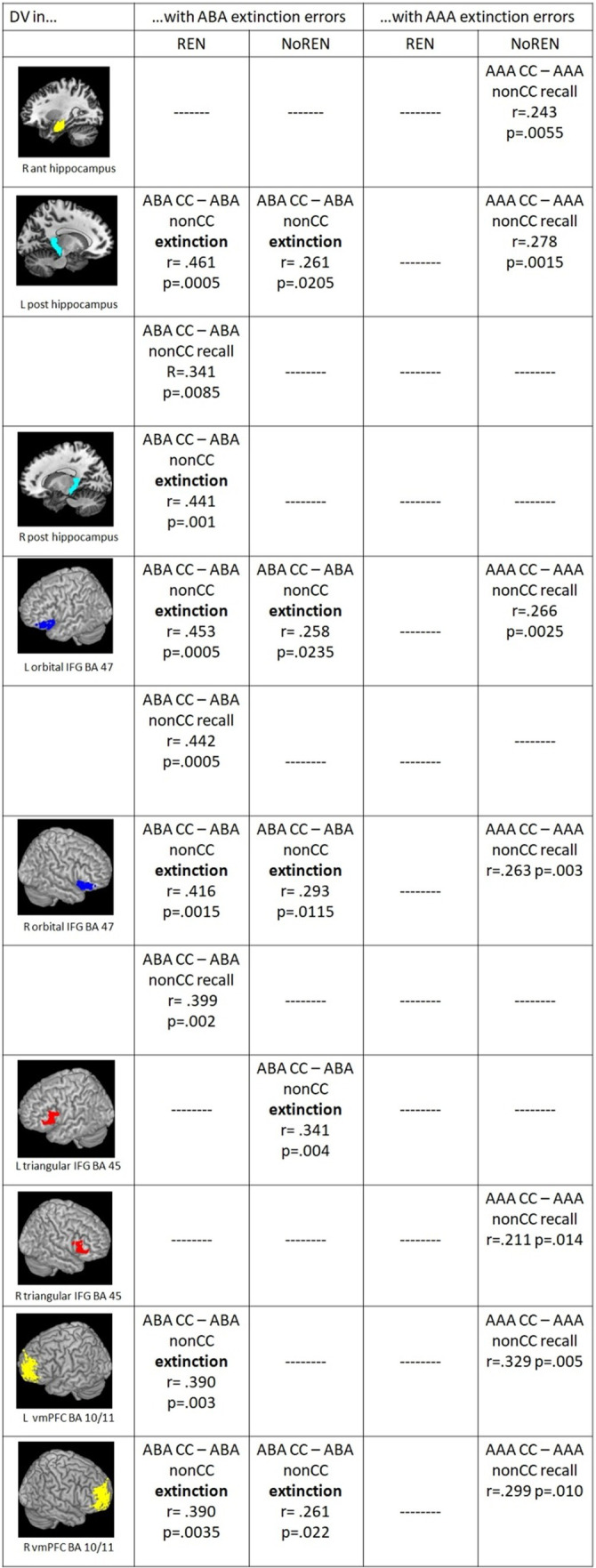
Significant correlations between ABA/AAA extinction errors and outcome-related DVs of ABA CC–ABA nonCC/AAA CC–AAA nonCC extinction/recall trials in the REN and NoREN groups (Pearson’s correlation coefficient, one-tailed).

In correlations of outcome-related DVs and extinction errors, the groups show commonalities as well as differences: Both groups exhibit significant positive correlations of ABA extinction errors and outcome-related DVs of trials in a novel context (ABA CC–ABA nonCC) in bilateral orbital IFG BA 47, left posterior HC and right vmPFC during extinction learning, In addition, only REN but not NoREN shows such significant correlations during extinction also in right posterior HC and left vmPFC, and during recall in left posterior HC and bilateral orbital IFG BA 47 (see Figure 5).

Further differences pertain largely to correlations of AAA extinction errors and trials in the identical context. Here, significant correlations occur only in the NoREN group, but not in REN: In NoREN we observed a number of significant positive correlations of AAA extinction errors with the outcome-related DVs of AAA CC-AAA nonCC during the recall phase. The result indicates that a higher level of AAA extinction errors in the extinction learning phase drives differentiation of outcomes in AAA trials during recall (but not during extinction learning proper) in HC, IFG, and vmPFC.

These results suggest that NoREN individuals focus more on extinction trials in an identical context than REN do, thus benefitting more from AAA extinction errors with regard to outcome-related discrimination of AAA trials during recall. In contrast, REN appears to focus stronger on the outcome-related discrimination of trials in a novel context (ABA CC–ABA nonCC), as shown by their positive correlations in various regions that are absent in NoREN.

Taken together, the findings suggest different foci of attention in the REN and NoREN groups: While NoREN responds with a higher level of attention to a change of outcome in an otherwise unchanged context-cue compound, REN tends to focus on the presence of a novel context.

#### 3.2.6 Correlations between behavioral measures

In the complete group (i.e., all REN and NoREN participants), ABA extinction errors correlated positively with AAA extinction errors (*r* = 0.467, *p* = 0.000) and AAA recall errors (*r* = 0.228, *p* = 0.017) but not with the ABA renewal level (*r* = −0.040, *p* = 0.682).

In the REN group, we also found a positive correlation of ABA extinction errors with AAA extinction errors (*r* = 0.401, *p* = 0.004), but not with AAA recall errors (*r* = 0.182, *p* = 0.210). Instead, REN shows a negative correlation between ABA extinction errors and ABA renewal level (*r* = −0.301, *p* = 0.035), indicating that in the subgroup of individuals with a propensity for renewal, higher error rates are linked to less renewal.

Considering these findings together with the negative correlation between context-related DVs and ABA renewal, as well as the positive correlation between context-related DVs and ABA extinction errors, we can assume that ABA extinction errors support a better discrimination of trials with and without a novel context. Moreover, a better discrimination of trials with and without a novel context reduces ABA renewal.

In the NoREN group, we too observed a positive correlation of ABA extinction errors with AAA extinction errors (*r* = 0.530, *p* ≤ 0.0001) and with AAA recall errors (*r* = 0.371, *p* = 0.003), but no significant correlation with ABA renewal level (*r* = 0.240, *p* = 0.065).

In the complete group, moreover, there is a significant positive correlation between ABA renewal and AAA recall errors: *r* = 0.287, *p* = 0.002. In the subgroups only, correlations were not significant (REN: *r* = 0.032, *p* = 0.829; NoREN: *r* = 0.205, *p* = 0.116).

#### 3.2.7 Group comparisons of behavioral measures

Renewal and NoREN did not differ significantly in their ABA extinction error level *t*(107) = 0.618, *p* = 0.538 (mean REN 18.82% ± 2.17 sem; mean NoREN 17.18% ± 1.61 sem), nor in their AAA extinction error level *t*(107) = 1.471 *p* = 0.144 (mean REN 20.31% ± 1.70 sem, mean NoREN 16.75% ± 1.68 sem).

By definition, the groups differed in their ABA renewal level *t*(107) = 20.079, *p* = 0.000 (mean REN 67.95% ± 3.68 sem, mean NoREN 0.83% ± 0.36 sem).

Also in the retrieval of AAA extinction memory during the recall phase we observed significant group differences, with more errors in the REN group *t*(107) = 3.300, *p* = 0.001 (mean REN 12.45% ± 3.08 sem, mean NoREN 2.5% ± 1.05 sem). Thus, responding during recall with the first-learned association (correct in the acquisition phase) was more pronounced in the REN group not only for ABA trials, but also for AAA trials, suggesting that next to actually context-based ABA renewal responses, impaired extinction memory may have contributed to a certain part of renewal responses (see Table 3).

**Table 3:** Learning performance of the REN and NoREN subgroups–percentage of errors resp.

Group	ABA extinction errors %	AAA extinction errors %	ABA renewal %	AAA recall errors %
REN	18.82 ± 2.17	20.31 ± 1.70	67.95% ± 3.68	12.45 ± 3.08
NoREN	17.18 ± 1.61	16.75 ± 1.68	0.83 ± 0.36	2.50 ± 1.05
Two-sample *t*-test	T(107) = 0.618, *p* = 0.538	T(107) = 1.471, *p* = 0.144	T(107) = 20.079, *p* = 0.000^***^	T(107) = 3.300, *p* = 0.001^**^

ABA renewal responses, ± standard error of means (s.e.m.).**statistically significant p<0.01.***statistically significant p<0.001.

## 4 Discussion

We analyzed potential group differences of dissimilarities in neural representations of context- and outcome-related trials in brain regions involved in extinction and renewal (HC, IFG, and vmPFC), as well as the relationship between dissimilarities and behavioral performance in individuals showing or not showing renewal. The main findings were as follows:

REN and NoREN differ markedly in their processing of extinction learning, regarding the extent to which extinction errors influence the ability to discriminate between trials.
In particular, REN and NoREN both show significant correlations of ABA extinction errors with context- and outcome-related DVs to varying degrees, but only NoREN also shows significant correlations of AAA extinction errors with context- and outcome-related DVs.
However, the general level of dissimilarities between neural representations of trials does not differ between REN and NoREN, with the exception of right posterior HC.
Moreover, in REN, correlations between ABA renewal and context-related DVs in IFG and HC regions are negative, indicating that higher dissimilarity of neural representations, reflecting better discrimination performance, is linked to less renewal.

### 4.1 ABA extinction errors support context-related and outcome-related discrimination in both groups

Two regions showed results that were common to REN and NoREN participants alike: in left posterior hippocampus and bilateral orbital IFG (BA 47), context-related and outcome-related discrimination were associated with ABA extinction errors in both groups. Probably, these regions support a mode of processing that is not necessarily related to renewal, but provide information for a response decision that may or may not result in renewal. Such a processing mode is in line with studies implicating IFG in selection from competing response options (Budhani et al., 2007; Mitchell et al., 2009).

In particular, positive correlations between ABA extinction errors and context-related DVs during recall indicate that more errors in extinction trials with a novel context support context-related discrimination in bilateral orbital IFG during later recall in both groups alike. In left posterior HC, positive correlations with DVs were observed during the extinction phase in REN, suggesting that REN uses context-related differentiation of trials already during extinction learning proper.

Also, REN and NoREN both show positive correlations between ABA extinction errors and outcome-related discrimination of ABA trials already during extinction learning proper, in left posterior HC, bilateral orbital IFG (i.e., the same regions as shown above for context-related discrimination), and—only in REN—also during recall.

By showing these correlations, the findings extend the fMRI results from the study of Lissek et al. (2020), from which a part of the datasets originated. This study found orbital IFG processing of extinction learning in both groups, with higher lefthemispheric activation in REN and higher righthemispheric activation in NoREN (Lissek et al., 2020). In a further study, there was a positive correlation between activation in left orbital IFG and ABA extinction errors (Klass et al., 2021).

Also, in both groups, vmPFC appears to support outcome-related discrimination of trials with a novel context, using the prediction error feedback from ABA extinction errors. However, in the REN group, vmPFC differentiation performance is linked to error feedback only in this analysis, while in NoREN, vmPFC is engaged in error feedback-related trial differentiation in all analyses, as will be described in a following paragraph. These results specify the role of vmPFC for extinction learning, and thus are consistent with a number of studies that implicated vmPFC in context-related extinction (Kalisch et al., 2006; Milad et al., 2007).

In further analyzed brain regions, relationships between context-related DVs in recall and ABA extinction errors were found either only in REN or only in NoREN, pointing toward differences in processing: on the one hand, bilateral vmPFC relationships were observed only in the NoREN group; and on the other hand, relationships in bilateral triangular IFG and right opercular IFG were observed only in the REN group. Opercular IFG activation in recall was previously found associated with a renewal effect, together with posterior hippocampus (Lissek et al., 2020), thus the correlation between ABA extinction errors and DVs in these regions during recall in the REN group indicates a processing mode probably supporting renewal.

Also regarding relationships between outcome-related DVs during extinction learning and ABA extinction errors, several regions showed correlations only in one of the groups: namely left vmPFC and right posterior HC (REN only), and left triangular IFG (NoREN only).

In summary, for both groups, prediction error processing, resulting from ABA extinction errors, apparently supports context-related discrimination performance between extinction trials with and without a novel context (ABA and AAA) during recall. During extinction learning, the prediction error guides attention towards the novel context (Darby and Pearce, 1995), a process which supposedly improves formation of context-related neural representations of extinction trials. While this phenomenon is present in both groups in bilateral orbital IFG and left posterior HC, additional participating brain regions differ between the groups, pointing toward differential processing strategies in REN and NoREN that may either promote or suppress renewal.

The findings support a role for vmPFC and orbital IFG in prediction error processing, complementing previous findings that found orbital and medial PFC involved in prediction error processing (Yang et al., 2023). The results are also in line with a view that considers hippocampus as a module that processes novelty signals through being triggered by violations of predictions (Kumaran and Maguire, 2006, 2009) and that computes discrepancies of expected and actual reinforcement (Patt et al., 2022).

### 4.2 Only the NoREN group benefits from AAA extinction errors for context-related and outcome-related discrimination of trial types

Importantly, individuals with and without renewal propensity differ in the processing of AAA extinction errors. Only NoREN shows significant positive correlations between context- or outcome-related DVs on the one hand, and AAA extinction errors on the other, in bilateral orbital IFG BA 47, and bilateral vmPFC.

In addition, AAA extinction errors are linked with only context-related DVs in bilateral triangular and opercular IFG, as well as in left anterior HC. Moreover, they are linked with only outcome-related DVs in right anterior and left posterior HC.

These findings indicate that in the NoREN group, errors in trials that are performed in an identical context support discrimination learning of trials that differ in their context as well as in their outcome. Despite similar AAA extinction error rates in both groups, such a relationship is completely lacking in REN individuals, and is therefore unique for NoREN individuals.

Not only does the REN group show no benefit from AAA prediction error feedback for the discrimination of trials, they even show significantly more AAA extinction recall errors in the recall phase than NoREN, indicating an inferior extinction memory in AAA trials.

While for REN, the novel context appears to be much more important for discrimination learning, for NoREN, discrimination learning appears to rely on both types of errors, ABA and AAA, with a somewhat more pronounced focus on AAA errors. These findings too point towards important differences in extinction processing of REN and NoREN individuals for these trial types.

Overall, the results suggest a higher level of attention to the novel context in REN individuals, which is in line with the assumption that renewal results from processing the unexpected change in context (Darby and Pearce, 1995).

### 4.3 vmPFC is involved in context-related and outcome-related differentiation only in NoREN

A further interesting difference between the groups pertains to the involvement of vmPFC. In NoREN, bilateral vmPFC is consistently involved in context- and outcome-related differentiation of trials, supported by ABA and AAA extinction errors, during recall and extinction. Positive correlations are observed between DVs of outcome-related trials in the identical context during recall and AAA extinction errors; as well as positive correlations of outcome-related trials in the novel context during the extinction phase proper and ABA extinction errors. In addition, context-related DVs during recall are positively correlated with both ABA and AAA extinction errors.

In contrast, in REN, right vmPFC is only involved in outcome-related discrimination of trials in a novel context during extinction, supported by ABA extinction errors.

It has been supposed that vmPFC has a major role in reward processing and value-based decision making in various tasks (Hiser and Koenigs, 2018; Rolls, 2022). Our results suggest that in the two groups, this processing mode in vmPFC is differentially associated with extinction errors. In NoREN, vmPFC discrimination abilities are more pronouncedly associated with extinction learning feedback for both outcome- and context-related DVs, furthermore the contributing extinction learning feedback is derived from both conditions.

Consequently, for the processing of reward and values, NoREN appears to command a wider base of examples to rest their decision upon; therefore processing in vmPFC may contribute to their lower error rates in specifically AAA recall, and also to the absence of ABA renewal.

In contrast, as mentioned above, the involvement of vmPFC in REN—restricted to outcome-related DVs in ABA trials—may promote renewal. In view of the proposed crucial role of vmPFC for reward processing and value-based decision making (Hiser and Koenigs, 2018; Rolls, 2022), REN, in comparison to NoREN, shows a relatively low level of vmPFC differentation based on extinction error feedback. Yet, in REN, but not in NoREN, vmPFC involvement was previously found during recall of ABA extinction compared to AAA, its activation positively correlated with ABA renewal (Lissek et al., 2013), as well as in a within-subject comparison of a recall phase with ABA renewal to one without renewal (Lissek et al., 2020). Thus, vmPFC in REN is active in recall, and—according to this RSA analysis—probably so by differentiating trials based on outcome rather than on contexts, a manner of processing that may potentially facilitate a renewal response.

### 4.4 Outcome-related discrimination as differentation between extinguished and unextinguished stimuli

In terms of extinction learning, outcome-related discrimination can be framed as a differentiation between extinguished and unextinguished stimuli (i.e., extinction stimuli and retrieval stimuli in our task).

As such, the results for outcome-related DVs in the AAA condition in the NoREN group for vmPFC extend the findings by Milad et al. (2007), who observed stronger activation in vmPFC during extinction recall for extinguished versus unextinguished stimuli. Our study thus shows that prediction error feedback from wrong responses during extinction learning apparently drives the discrimination, so that higher error rates contribute to a better discrimination in vmPFC. The results are in line with findings that showed vmPFC activation associated with prediction error signalling in human fear conditioning (Spoormaker et al., 2011). Moreover, in the ABA condition, such a differentiation between extinguished and unextinguished stimuli already occurs during the extinction phase proper: namely, in bilateral vmPFC in the REN group and in right vmPFC in the NoREN group. Probably, the presence of a novel context speeds up the differentiation between stimuli that change their consequence and stimuli that do not.

Also bilateral orbital IFG (BA 47) shows outcome-related discrimination correlated with ABA extinction errors in both groups. Additionally, in NoREN, this region also contributes to discrimination in context- and outcome-related trials, supported by the prediction error feedback from AAA extinction errors.

Conceivably, therefore, orbital IFG is involved in processing the distinction between extinguished and unextinguished trials, no matter whether extinction occurs in a novel or the identical context. This would be in line with the supposed role of IFG in processing competing response options (Novick et al., 2005; Budhani et al., 2007; Mitchell et al., 2009) and its involvement in sequence processing (Planton and Dehaene, 2021): in AAA trials response options differ only with regard to their temporal sequence, while in ABA trials, response options can additionally be distinguished by way of the context of extinction. Accordingly, orbital IFG appears also involved in processing the distinction between novel and same contexts; therefore, context information also appears to have a role when processing competing response options.

The contribution of posterior HC regarding outcome-related discrimination presumably consists in providing contextual information for vmPFC and IFG processing.

### 4.5 Differences in discrimination performance in HC between REN and NoREN

The only region in which REN and NoREN differ significantly in their levels of context-related DVs is right posterior HC, where REN shows stronger context-related discrimination during extinction learning. This result extends previous findings that regularly observed higher BOLD activation in posterior HC in REN. The new findings suggest that posterior HC actually supports processing information regarding the novel context, and may aid in distinguishing trials that contain this changed context from those that do not.

Previous studies could only show that in REN, ABA extinction yielded higher activation than AAA extinction in (bilateral) posterior HC, which was not the case in NoREN (Lissek et al., 2013). Posterior hippocampal activation was also found already during acquisition in REN participants, and during processing of both the context presented alone, and presentation of context and cue together (Lissek et al., 2016). Within-subject comparisons during a switch from no renewal to renewal also showed a contribution of bilateral posterior HC during recall that resulted in renewal (Lissek et al., 2020).

The present results suggest that the REN group‘s higher activation in posterior HC is presumably linked to their increased context-related discrimination, which, however, was found inversely related to renewal, as will be discussed in the following paragraph.

In both groups, predominantly left posterior HC benefits from ABA prediction error feedback for differentiation of trials based on context and outcome. Only in NoREN, also anterior HC benefits from AAA prediction error feedback for differentiation of trials based on context and outcome. The participation of anterior HC, together with more pronounced participation of vmPFC for using AAA error feedback in NoREN may reflect the stronger connectivity of anterior HC with vmPFC, as opposed to the stronger connectivity of posterior HC with IFG, found in a recent study (Frank et al., 2019).

The results suggest that next to processing context, HC also has a role in processing outcomes, which is in line with findings that show increased hippocampal activation after violation of predictions (Kumaran and Maguire, 2006; Bein et al., 2020). Taking cues from prediction errors, HC is assumed to update memories by incorporating relevant details from recent experiences (Sinclair et al., 2021), a function which may link context and outcome processing in HC in the task used in this study.

### 4.6 In the REN group, better context-related discrimination during extinction learning is associated with less ABA renewal

In contrast to our hypothesis, however, better discrimination of trials with and without a novel context during extinction learning was not associated with more ABA renewal, but instead with less ABA renewal during recall. This relationship was observed in many of the studied regions: in bilateral posterior HC, bilateral triangular IFG BA 45 and right opercular IFG BA 44. The effect is predominantly based on the correlations of those REN participants with higher ABA renewal levels (70–100%), indicating that it is linked to an actual processing strategy, and not to a comparably rather random response behavior.

Thus, participants who show higher context-related discriminative ability of ABA and AAA trials during extinction learning—i.e. during the phase when the surprising change of outcome is assumed to direct attention to the context (Darby and Pearce, 1995)—exhibit less ABA renewal during recall, indicating that better formation of context-related representations in the above regions promotes a lower degree of renewal. Vice versa, this finding suggests that a high level of ABA renewal is associated with less stable representations.

Since context-related DVs in IFG and posterior HC are higher in REN individuals who show less renewal, the context-related discrimination of extinction trials may be a factor contributing to renewal, but is obviously not a crucial factor.

A finding supporting this assumption is that context-related discrimination abilities appear to be similar in individuals showing and not showing renewal, since the DVs in most areas (with the exception of right posterior HC), do not differ significantly between REN and NoREN participants. Therefore, a potential contribution to renewal cannot possibly be based on the absolute level of DVs, but presumably rather depends on how the information contained in these context-related neural representations is further processed in the network.

Particularly right posterior HC, where REN shows significantly better discrimination performance, is a candidate region for contributing to contextual discrimination. The finding of differential discrimination performance in REN and NoREN in thie region is in line with a number of previous studies that observed higher activation in posterior HC during extinction learning and recall in REN compared to NoREN and suggested that the contribution of hippocampus to renewal consists in providing context information (Lissek et al., 2013, 2016, 2020). Our present results extend these findings by showing that posterior HC can actually discriminate between contexts, and that this discrimination potential is higher in REN than in NoREN.

However, also in right posterior HC, the correlation between ABA renewal level and context-related DVs for discrimination of extinction trials is negative. Therefore, the information proper provided by posterior HC does not determine whether renewal occurs.

Also in bilateral IFG, the correlation between ABA renewal level and context-related DVs is negative. Thus, better discrimination of ABA and AAA extinction trials also in this region, which is involved in processing of competing response options (Novick et al., 2005; Budhani et al., 2007; Mitchell et al., 2009), promotes less ABA renewal during recall. The task of processing competing response options presumably also requires processing contextual information since these may differ between response options.

Interestingly, we found no significant negative correlation between ABA renewal and context-related DVs during recall proper, when the decision for a renewal response is actually made. However, there are three regions which exhibit a trend toward a significant negative correlation during recall (R ant HC: *r* = −0.250, *p* = 0.085. L BA 47 *r* = −0.249, *p* = 0.085, R BA 47 *r* = −0.241, *p* = 0.095). Therefore, while we cannot claim that worse discrimination during recall proper is linked to more renewal, there are hints that point into this direction.

## 5 Conclusion

Overall, the findings demonstrate that the absolute level of discrimination between trial types on the basis of context or outcome has no prominent impact upon the propensity to show renewal. The only region where participants with renewal showed a higher level of context-related discrimination was right posterior HC, a result which, consistent with previous findings, points towards an involvement of this region in renewal.

In addition, the NoREN group shows an overall stronger link between extinction errors and context- and outcome-related DVs, integrating error feedback from both ABA and AAA trials into their discrimination performance, while REN shows no link between AAA errors and discrimination at all. Presumably, the processing strategy of the NoREN group may support a response selection that does not result in renewal.

Importantly, in the REN group, a higher ABA extinction error rate promoted better context-related discrimination of extinction trials, which in turn was linked to less ABA renewal. Thus, ABA renewal presumably does not implicate particularly good context-related discrimination of trials, but may instead reflect inferior discrimination of trial types.

## Data availability statement

The raw data supporting the conclusions of this article will be made available by the authors, without undue reservation.

## Ethics statement

The studies involving humans were approved by Ethics board of the Faculty of Medicine of Ruhr University Bochum, Germany (Reg.No. 3022-10). The studies were conducted in accordance with the local legislation and institutional requirements. The participants provided their written informed consent to participate in this study.

## Author contributions

SL: Conceptualization, Formal analysis, Funding acquisition, Methodology, Writing – original draft, Writing – review & editing, Supervision. MT: Funding acquisition, Project administration, Supervision, Writing – review & editing.
